# Signal-to-Noise Ratio Measures Efficacy of Biological Computing Devices and Circuits

**DOI:** 10.3389/fbioe.2015.00093

**Published:** 2015-06-30

**Authors:** Jacob Beal

**Affiliations:** ^1^Raytheon BBN Technologies, Cambridge, MA, USA

**Keywords:** synthetic biology, controls, signals, digital circuits, Boolean logic, analysis

## Abstract

Engineering biological cells to perform computations has a broad range of important potential applications, including precision medical therapies, biosynthesis process control, and environmental sensing. Implementing predictable and effective computation, however, has been extremely difficult to date, due to a combination of poor composability of available parts and of insufficient characterization of parts and their interactions with the complex environment in which they operate. In this paper, the author argues that this situation can be improved by quantitative signal-to-noise analysis of the relationship between computational abstractions and the variation and uncertainty endemic in biological organisms. This analysis takes the form of a ΔSNR_dB_ function for each computational device, which can be computed from measurements of a device’s input/output curve and expression noise. These functions can then be combined to predict how well a circuit will implement an intended computation, as well as evaluating the general suitability of biological devices for engineering computational circuits. Applying signal-to-noise analysis to current repressor libraries shows that no library is currently sufficient for general circuit engineering, but also indicates key targets to remedy this situation and vastly improve the range of computations that can be used effectively in the implementation of biological applications.

## 1 Introduction

Engineering biological cells to perform computations has been one of the major goals of synthetic biology from its inception (Knight and Sussman, 1998; Elowitz and Leibler, 2000; Gardner et al., 2000; Weiss, 2001). The complexity of computations that have actually been implemented, however, has been quite small (Purnick and Weiss, 2009), only quite recently rising as high as a 3-layer logic circuit comprising 6 regulatory devices (Moon et al., 2012). A number of well-known obstacles have contributed to the difficulty of building multi-element logic circuits, including insufficient numbers of strong regulatory elements for building circuits, undesirable interactions between genetic elements, difficulties in constructing and delivering large genetic constructs, and difficulty in modeling and predicting circuit behavior. A number of ongoing efforts are showing progress toward decreasing these problems in circuit engineering, promising to soon deliver many more strong regulatory elements [e.g., Bonnet et al. (2013), Kiani et al. (2014), and Stanton et al. (2014)], improved isolation between components [e.g., Lou et al. (2012) and Mutalik et al. (2013)], fast and easy construction and delivery [e.g., Weber et al. (2011) and Linshiz et al. (2012)], and better predictive circuit models [e.g., Davidsohn et al. (2015) and Beal et al. (2015)].

Among all of these improvements in our ability to engineer computational circuits, however, there are two critical and surprisingly unresolved questions:

- Just how good an implementation of computation is provided by the biological circuits and genetic elements that are currently available?
- How much better do they need to be in order to realize various applications?

A number of efforts have been made toward providing a clear definition for biological computational devices [e.g., Knight and Sussman (1998) and Weiss (2001)] and toward characterizing their performance [e.g., Canton et al. (2008), Ellis et al. (2009), Kelly et al. (2009), and Beal et al. (2012)]. None of these efforts to date, however, has provided a practical method for quantifying the performance of real devices and circuits that can be implemented with readily obtainable information about biological devices.

This paper aims to provide such a method, based on the mathematical foundation of a signal-to-noise ratio (SNR). The basic idea is this: although biological computation is defined in the platonic realm of abstract numbers and symbols, it must be realized in the noisier physical reality of quantities like chemical concentration. Such reality is never perfect, and a signal-to-noise ratio quantifies how much of a problem the noise is with respect to the intended representation. In electronics, signal-to-noise analysis is a foundational tool for the engineering of computation and communication; this paper now adapts this tool to the engineering of biological computation circuits.

To this end, Section 2.1 of this paper thus begins by reviewing these foundational concepts and adjusting their application to be suitable for biological circuits. Section 2.2 applies this to computing devices, analyzing them in terms of the degree to which they enhance or degrade signal strength under various conditions. Section 2.3 shows how SNR analysis of individual devices can be used to predict the behavior of circuits, and Section 3.1 follows the implications of these methods to develop a new framework for engineering biological circuits based on SNR analysis. Building on this framework, Section 3.2 applies SNR analysis to existing libraries of biological computational devices, finding that none are yet suitable for large-scale circuit engineering and identifying targets for improvement that may remedy this situation. Finally, Section 4 summarizes and considers future directions.

## 2 Materials and Methods

### 2.1 Boolean biochemical signals

For any biochemical implementation of Boolean values, we need to choose what physical phenomena will be interpreted as the abstract values “true” and “false.” In this paper, we will focus on one of the earliest proposed (Knight and Sussman, 1998; Weiss, 2001) and most commonly used representations, in which Boolean values are represented by the concentration of particular chemical species within a cell.

Many other biological phenomena have also been proposed or used to represent Boolean values, including extracellular concentration of chemicals [e.g., Danino et al. (2009)], rate of transcription by DNA polymerase or translation by ribosomes [e.g., Canton et al. (2008)], presence, absence, or inversion of a given DNA sequence [e.g., Bonnet et al. (2013)], epigenetic markings on a DNA sequence [e.g., Keung et al. (2014)], fluorescence or light emission [e.g., Kim and Lin (2013)], and trans-membrane voltage [e.g., Adams and Levin (2013)]. For nearly all such mechanisms of biological computation, however, at some point the coupling between elements in the computation is regulated by the concentration of some chemical species. Thus, for many of these alternative representations of Boolean values, it is possible to identify an equivalent chemical representation to which the signal analysis developed in this paper can be applied.

We can evaluate the quality of a chemical concentration representation of Boolean values by comparing the distribution of concentrations per cell produced when the chemical should be in the “true” state with the distribution of concentrations per cell when the chemical should be in the “false” state. The more that these two distributions overlap, the harder it is to distinguish between them, and therefore the worse the quality of the signal and the more difficult it is to engineer an effective computation. Likewise, the more that the two distributions they are separated, the better the quality and the easier it is to engineer.

In electromagnetic systems, this notion of quality is typically quantified as a signal-to-noise ratio (SNR).^1^ Signal-to-noise ratio is normally measured on a logarithmic scale of decibels, which can be computed using the standard definition:

(1) $${\text{SNR}}_{\text{dB}}=20\;\log_{10}\frac{A_{\text{signal}}}{A_{\text{noise}}}$$

where *A* is the root-mean-square (RMS) amplitude of the signal and noise waveforms, respectively (Oppenheim and Willsky, 1997). Applied to a general Boolean signal, this becomes:

(2) $${\text{SNR}}_{\text{dB}}=20\;\log_{10}\frac{\left|\mu_{\text{true}}-\mu_{\text{false}}\right|}{2\sigma }$$

computing expected signal amplitude as half the difference between mean “true” value and the mean “false” value (i.e., approximated by the RMS amplitude of a square wave), and noise amplitude as σ, the mean standard deviation for true and false states (i.e., the RMS amplitude for the waveform remaining when the intended Boolean signal is subtracted).

Superficially, it seems that the same analysis should be applicable to biochemical systems. In fact, however, this is not the case. The problem is that strong cellular expression of chemicals typically exhibits a log-normal distribution of concentration per cell [see, e.g., Friedman et al. (2006), Beal et al. (2012), Bonnet et al. (2013), Davidsohn et al. (2015), and Stanton et al. (2014)] – even if output might be population-level, computation is currently typically carried out within individual cells, because there are currently many more intracellular than intercellular devices available. This means that both signal and noise are generally better represented using geometric statistics, implying that the signal-to-noise ratio calculation becomes:

(3) $${\text{SNR}}_{\text{dB}}=20\;\log_{10}\frac{\left|\log_{10}\left(\mu_{g,\text{true}}∕\mu_{g,\text{false}}\right)\right|}{2\cdot \log_{10}\left(\sigma_{g}\right)}$$

where the μ_*g*_ variables are the geometric means of the true and false values and σ_*g*_ is the geometric standard deviation for both states (i.e., variation expressed in fold times/divide rather than value plus-minus).

The SNR that is actually required depends on the application. For example, if the goal is simply to detect that a computation is followed a specified truth table, this can be accomplished even when the signal is significantly less than the noise. For example, achieving a twofold difference in signal levels in a system with twofold SD of noise requires only an SNR_dB_ of only $20\;\log_{10}\frac{\left|\log_{10}\left(2\right)\right|}{2\cdot \log_{10}\left(2\right)}=-6.0\text{dB}$. For another example, controlling cells in an industrial fermenter, such that they select the most efficient of two modes of operation based on changing local conditions, are still a fairly permissive application, since individual cells selecting the wrong choice is likely to have only a minor effect on the overall batch process, and thus might require a fairly low SNR, in the 0–5 dB range. At the opposite end of the scale, a system intended to identify and kill cancer cells inside of a human patient likely needs to have a much higher SNR, perhaps in the range of 20–30 dB, since even a small fraction of cells erroneously killing healthy cells may have a major adverse impact on the patient’s health.

**Figure 1:**
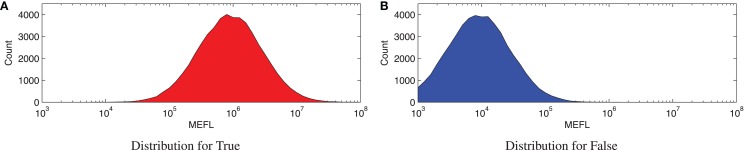
**Example of a Boolean signal with values that cannot be perfectly distinguished: (A,B) show histograms for 50,000 cells sampled from typical distributions for Boolean true and false states, respectively**. These distributions overlap, however, and in the overlapping range it is difficult or impossible to distinguish between true and false values.

For an example of such an SNR calculation, consider the simulated distributions shown in Figure 1. These distributions are generated from a log-normal process using values drawn from within the typical range of expression in mammalian cells, as based on the experimental data in (Beal et al., 2012, 2015; Kiani et al., 2014; Davidsohn et al., 2015): μ_*g,*true_ = 10^6^ molecules of equivalent fluorescein (MEFL),^2^ μ_*g,*false_ = 10^4^ MEFL, and σ_*g*_ = 3.2-fold. Each distribution shown is a 10 bins/decade histogram of expressed fluorescence from 50,000 simulated cells.

Here, Figure 1A shows high expression representing a true state, while Figure 1B shows low expression representing a false state. The geometric means of these two distributions are nicely separated, with an approximately 100-fold ratio between the true and false levels. The cell-to-cell variation, however, is also fairly strong, with a σ_*g*_ of more than threefold, resulting in an overall SNR of only 6.2 dB.

Notice that the SNR value here is not very high, due to the high degree of cell-to-cell variation. Such relatively low SNRs are unfortunately rather typical for biological systems, and are an important factor in the difficulty of engineering reliable biological computations. The consequence is a low margin for error in design, putting even more importance on the quality of computing elements.

### 2.2 Effects of computation on signal strength

Each computational element in a biological circuit, in addition to performing its intended purpose, also affects the signal-to-noise characteristics of the signals passing through it. An element with strong amplification and inputs that are well-matched to its range of operation will produce *true* and *false* outputs that are more distinct than the inputs, i.e., with an increased SNR. An element with poorly matched inputs or poor amplification, on the other hand, will produce outputs that are less distinct than the inputs, i.e., with a decreased SNR. We may thus summarize the “quality” of a computational element in terms of the difference between input SNR and output SNR across the various combinations of inputs with which it may be supplied:

(4) $$\Delta {\text{SNR}}_{\text{dB}}={\text{SNR}}_{\text{dB,output}}-\;{\text{SNR}}_{\text{dB,input}}$$

Under this definition, the higher the ΔSNR_dB_, the better the biological element is at implementing a computation.

In general, the effect of a computational element is on SNR is not uniform, but depends on the circumstances of its use. This fact is independent of any additional biological effects of context, such as metabolic competition, toxicity, or translational read-through. Rather, it is an inherent characteristic of the non-linear relationships between input and output found in most computational elements: different combinations of input levels and noise environment (μ_*g,*true_, μ_*g,*false_, and σ_*g*_) produce different output SNRs. The output SNR is also affected by the dynamics of a signal, e.g., how often the value of the input changes. For the analysis in this paper, however, we will focus only on converged behavior in response to a stable input.^3^

**Figure 2:**
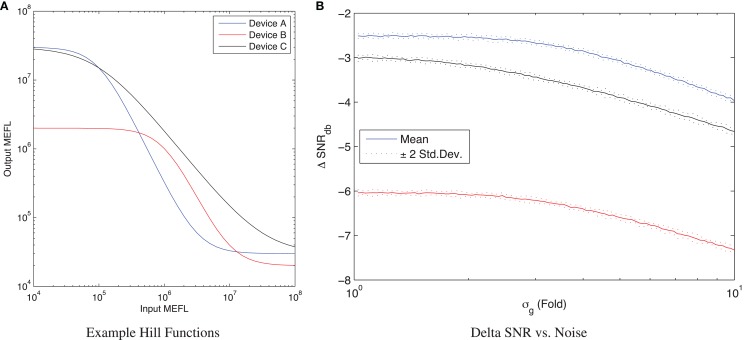
**The input/output curve of a device can be used to analyze its ΔSNR_dB_**. **(A)** Three example input/output curves, Device A (blue) is stronger than Device B (red) and also has more similar input and output ranges, while Device C (black) has a similar range to Device A but flatter slope. **(B)** For any given input levels, the observed ΔSNR_dB_ depends on the amount of noise σ_*g*_, converging to a maximum at low noise and falling as the noise increases.

The ΔSNR_dB_ for a computational element can be computed from an input/output curve, i.e., a function measuring the outputs observed across a range of input levels. Figure 2A shows three simulated examples of such curves for repressor devices with one input and one output (note that to allow easy visualization, all examples in this paper will be restricted to one input and one output, but the methods presented work for multiple inputs and multiple outputs as well). The three example devices have input/output curves *f_i_* generated using Hill equations (Hill, 1910)^4^ of the form:

$$\mathrm{Out}=\alpha \cdot \frac{1+K^{-1}{\left(\frac{\mathrm{In}}{D}\right)}^{H}}{1+{\left(\frac{\mathrm{In}}{D}\right)}^{H}}$$

with parameters selected to place the curve within the typical observed range for current repressor devices (Bonnet et al., 2013; Kiani et al., 2014; Stanton et al., 2014; Davidsohn et al., 2015), and both *In* and *Out* concentrations expressed in MEFL. In particular, Device A (blue) uses *K* = 10^3^, *D* = 10^5^, *H* = 2, α = 3 × 10^7^, while Device B (red) uses *K* = 10^2^, *D* = 10^6^, *H* = 2, α = 2 × 10^6^, and Device C (black) uses *K* = 10^3^, *D* = 10^5^, *H* = 1.2, α = 3 × 10^7^. As can be seen in Figure 2A, Device A has a larger range than Device B, though their slope is similar in the transition between values; Device A also has a more similar range for its outputs and inputs. Device C, meanwhile, has similar input and output ranges to Device A, but a significantly flatter slope.

When the expression noise σ_*g*_ is low, the ΔSNR_dB_ for a computational element is converged to a maximum determined by the difference between input range and output range. At the opposite extreme, as σ_*g*_ continues to increase, the ΔSNR_dB_ decreases, eventually converging to a linear slope entirely dominated by noise. For example, Figure 2B shows ΔSNR_dB_ for the three example devices as a function of uncorrelated noise^5^ for inputs with μ_*g,*true_ = 10^8^ MEFL and μ_*g,*false_ = 10^4^ MEFL, simulating 10 samples of 50,000 cells per sample. Notice that as expression noise decreases toward the minimal-noise limit of σ_*g*_ = 1, the ΔSNR_dB_ converges to an upper limit of around −2.5 dB for Device A, −6 dB for Device B, and −3 dB for Device C. As the expression noise increases, the SNR degrades as the distributions become less separated. By σ_*g*_ = 3, the noise is having a noticeable effect on all three devices, and by σ_*g*_ = 10 it degrades device performance by around 1.5 dB for all three devices.

**Figure 3:**
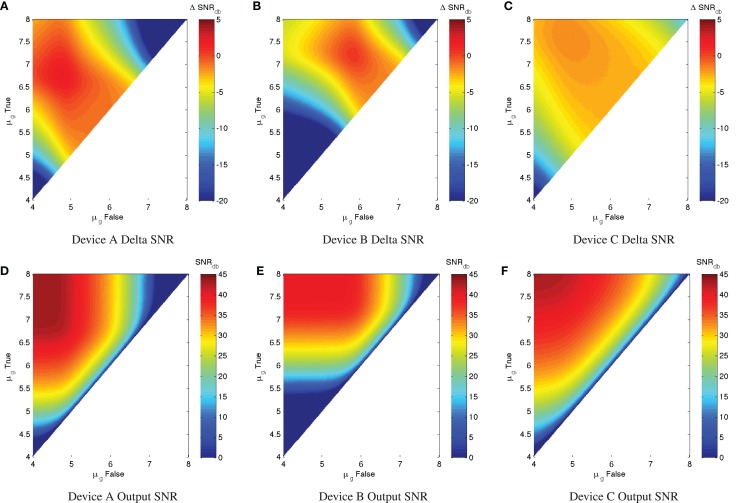
**Variation of maximum ΔSNR_dB_ and low-noise output SNR_dB_ (σ_*g*_ = 1.02) with respect to input levels for three example devices**. **(A–C)** show ΔSNR_dB_ for device A, B, and C, respectively, while **(D–F)** show low-noise output SNR_dB_ for the same devices. Note that the color scales are truncated at the lower end to provide better resolution in the upper range.

A good upper limit on the computational quality of a device can thus be estimated by considering the noise-free limit of its performance for various input levels. Figure 3 illustrates this with a simulation parameter scan of μ_*g,*true_ and μ_*g,*false_ for each example device. In specific, the scan simulates all combinations of μ_*g,*true_ > μ_*g,*false_ in the range of 10^4^–10^8^ MEFL in logarithmic steps at 50 levels/decade, for each combination running one sample of 50,000 cells at a very low noise σ_*g*_ = 1.02. Given the input/output functions for each of the example devices, the maximum output SNR at this noise level is 43.5 dB for Device A, 40 dB for Device B, and 43.1 dB for Device C. For each device, the strongest output SNR is found for input signals in the saturated regions of the device input/output curves: for Device A roughly μ_*g,*true_ > 10^6.5^ and μ_*g,*false_ < 10^5^, for Device B roughly μ_*g,*true_ > 10^7^ and μ_*g,*false_ < 10^6^, and for Device C roughly μ_*g,*true_ > 10^7.5^ and μ_*g*,false_ < 10^5^. At the boundary of this region, the strong slope of the Devices A and B allows some minor signal restoration, but outside of a relatively small “sweet spot” the output SNR degrades badly with respect to the input SNR. Device C has a similar “sweet spot” pattern, but its lower input/output curve slope means that even its best possible performance still sees a significant signal degradation ΔSNR_dB_ = −1.6dB.

Such a ΔSNR_dB_ chart can provide a good first analysis of the efficacy and operating range of a device. For example, with our three example devices, Device A has a decent range of potential use, while Device B is much narrower, and Device C, although has a very strong on/off ratio, significantly degrades signal strength even under ideal conditions of usage.

**Figure 4:**
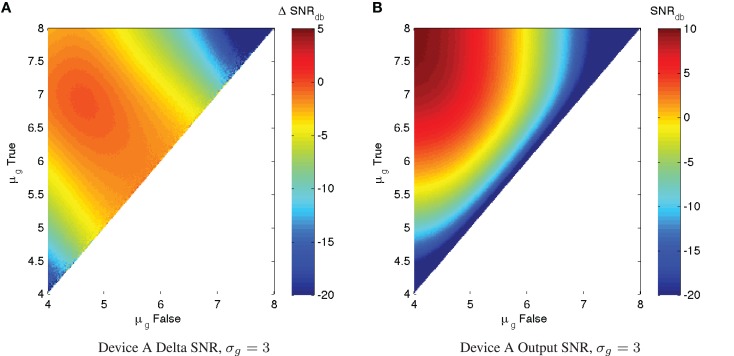
**With significant expression noise, ΔSNR_dB_ may be significantly worse than under ideal conditions**. For example, the charts above present the same analysis of Device A as in Figure 3, **(A)** shows ΔSNR_dB_, **(B)** shows output SNR_dB_, but with a more typical σ_*g*_ = 3 level of expression noise. Note that the color scales are truncated at the lower end to provide better resolution in the upper range.

In practice, of course, there is typically a significant level of expression noise, which further degrades the SNR characteristics of a device. With measurements of the expected σ_*g*_ for a device (which can be readily obtained through high-throughput per-cell assays such as flow cytometry or microscopy with automated image analysis), we can apply the same SNR analysis to estimate the actual expected ΔSNR_dB_, which will always be overall worse (more negative) than in the ideal minimal-noise condition. Figure 4 shows an example of such an analysis for Device A with σ_*g*_ = 3, a typical level of observed expression noise (simulated using the same parameters as before). Notice that the essential character of the chart is not changed, meaning that the conclusions drawn from the maximum SNR analysis still apply. All of the features of the SNR chart, however, are more “blurred,” degrading the regions of high performance. Ironically, this also somewhat mitigates the regions of worst performance, but performance in these regions is still generally too poor to be useful.

Computation of ΔSNR_dB_ can be used as a first stage of triage in analyzing whether a given biological device will be useful in attempting to realize digital computations. First, a device cannot be used at all unless it has both a ΔSNR_dB_ that is sufficient to meet application SNR requirements, and also can achieve that ΔSNR_dB_ requirement in a range matched with its inputs [such evaluation has the obvious pre-requisite of characterizing the input/output relation using SI units rather than relative units, e.g., by means of the protocols in Beal et al. (2012, 2015), Davidsohn et al. (2015), and Kiani et al. (2014)]. Beyond that, the wider the region of good ΔSNR_dB_, the easier it will be to match a device with others to form a circuit and the more tolerant a device will be of other types of perturbations inflicted by its context of deployment.

### 2.3 Multi-device computational circuits

Just as the computational efficacy of a single biological device may be analyzed in terms of its signal-to-noise characteristics, so can the same approach be applied to analyzing the computational efficacy of an entire computational circuit. The complete circuit can, after all, be viewed as just a more complicated device, and the SNR for its inputs and outputs be computed in the same way as for a single device.

The converged SNR characteristics of a circuit with no feedback loops can be predicted using the single-device SNR charts presented in the previous section. As has recently been demonstrated (Davidsohn et al., 2015), the mean and expression variation of such circuits can be predicted with high accuracy. Given such predictions, the maximum possible ΔSNR_dB_ can be predicted directly from the input signal levels, using the input-output curves and ΔSNR_dB_ analyses for the individual devices. For example, consider a chain of repressors, acting as logical inverters. For the *i*th inverter in the chain, its output is given by its input/output function *f_i_*, producing the input for the next stage. In the minimal-noise case, the SNR changes at each step are independent, meaning that they add linearly. The ΔSNR_dB_ for the circuit can thus be computed by composing together input-output curves to predict the inputs for each device, then summing for each device *i* the device ΔSNR_dB_*_,i_* along the path from input to output. This produces a total end-to-end change of:

(5) $$\begin{array}{l} \text{Δ }\underset{\text{dB,total}}{\text{SNR}}=\text{Δ }\underset{\text{dB,1}}{\text{SNR}}(\mu_{g\text{,true}},\mu_{g\text{,false}})+\text{ Δ }\underset{\text{dB,2}}{\text{SNR}}\;(f_{1}\;(\mu_{g\text{,false}}),\;f_{1}\;(\mu_{g\text{,true}}))\\ \;+\text{ Δ }\underset{\text{dB,3}}{\text{SNR}}\;(f_{2}\;\cdot \;f_{1}\;(\mu_{g\text{,true}}),\;f_{2}\cdot f_{1}\;(\mu_{g\text{,false}}))+... \end{array}$$

The overall efficacy of a circuit is thus a function of both the SNR properties of individual devices and how well signal levels are matched between devices. As seen in the previous section, positive SNR ranges may often be quite narrow, and even a relatively small mismatch can be disastrous for the efficacy of a computation.

**Figure 5:**
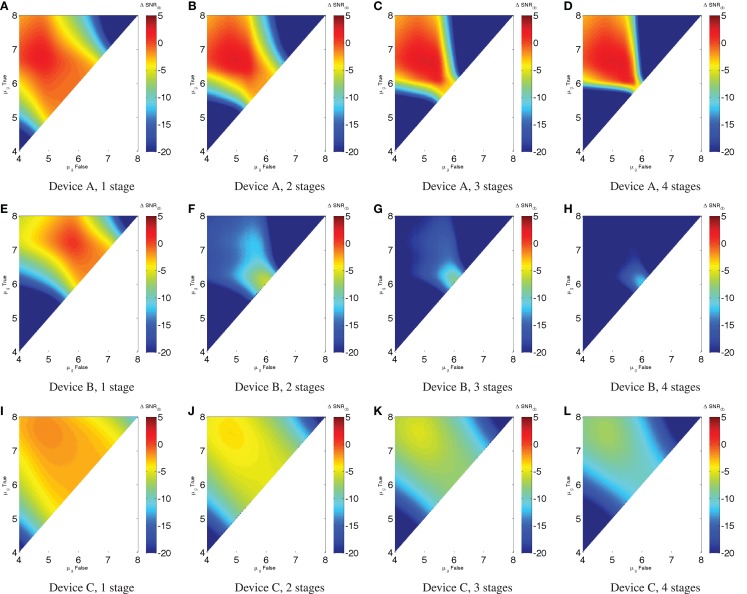
**Low noise ΔSNR_dB_ in a chain of inverters: (A–D) show chains of one to four Device A elements, (E–H) show chains of Device B elements, and (I–L) show chains of Device C elements**. Notice that for Device A there is a range of widely separated inputs (approximately μ_*g,*false_ < 10^5.5^, μ_*g,*true_ > 10^6^), where it is possible for SNR to remain strong; Device B is weaker and less well matched between input and output, and thus any computation with more than a single element has a badly degraded signal strength for all possible inputs. Device C, on the other hand, degrades incrementally in SNR across a broad range. Note that the color scales are truncated at the lower end to provide better resolution in the upper range.

For example, Figures 5A–D show the ΔSNR_dB_ for chains of one to four repressors, each with the characteristics of Device A. This is a nice example of (potentially) effective digital computation: Device A is strong enough to restore signal and a fairly good match between its input and output ranges. As a result, any input starting in a fairly broad region of the upper left can maintain a strong SNR over multiple stages of computation – in fact, an unbounded number in the absence of noise. Inputs falling outside of this good operating range, however, quickly degrade away to very low SNR.

This presages the problems that occur when the output and input levels of devices are not as well matched (or less strong, which makes for a smaller “sweet spot” and more difficulty in matching levels). For example, Figures 5E–H show chains of one to four repressors with the characteristics of Device B. Although its performance characteristics are not much worse than Device A for a single repressor (as seen in the previous section), the poor match with a narrow high-SNR “sweet spot” means that ΔSNR_dB_ collapses when a second repressor is added – much worse than the twice the original ΔSNR_dB_ – and continues to degrade thereafter. Indeed, the “least bad” region is where the high and low inputs hold almost the same value to begin with, meaning there is little signal to be lost in the first place.

With a good match between signal levels but not a steep enough slope of the input/output curve, there is a third mode of behavior. This is exemplified by Figures 5I–L, which show chains of one to four repressors with the characteristics of Device C. Without a region of positive ΔSNR_dB_, the signal cannot be sustained, but degrades incrementally. With devices of this sort, it is impossible to implement many-layer computations, but computations with only a few devices between any input and output are viable.

As with individual devices, of course, the minimal-noise model gives only a best-case evaluation of the computational efficacy of a circuit. This is still valuable, because it can be used to eliminate many non-viable options and to triage viable options based on the difficulty of attaining the (SNR_dB_ required for an application.

**Figure 6:**
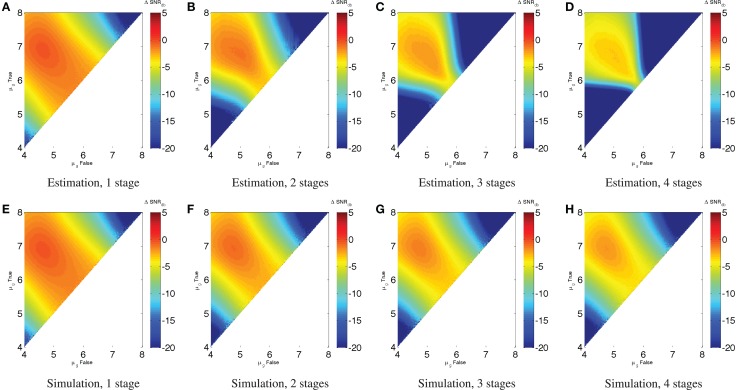
**The efficacy of a circuit with noisy distributions can be estimated from the (SNR_dB_ for individual devices under the same noise conditions**. For example, estimates of chains of Device A repressors with σ_*g*_ = 3 **(A–D)** are a good conservative bound on the behavior observed in simulation **(E–H)**. Note that the color scales are truncated at the lower end to provide better resolution in the upper range.

Just as with individual devices, however, we can use the same signal-to-noise models to estimate the performance of a circuit with higher *σ_g_*. As before, the best (SNR_dB_ is expected to be less than can be achieved in a minimal-noise circuit, though some of the worst performance can be mitigated. Unlike the minimal-noise case, however, we cannot precisely predict performance of the circuit by adding single-device SNR losses. At higher levels of expression noise, SNR losses are not independent because the operation of each device affects the effective *σ_g_* observed by the devices that consume its output. We can, however, estimate a conservative lower bound on performance by adding single-device SNR losses. For example, Figure 6 shows (SNR_dB_ for the Device A repressor chains with *σ_g_* = 3. Figures 6A–D estimate the value from the (SNR_dB_ of Device A with *σ_g_* = 3 shown in Figure 4A, while Figures 6E–H simulate chains of Device A using the same parameters as in Section 2.2 (*K* = 10^3^, *D* = 10^5^, *H* = 2, α = 3 × 107, σ_*g*_ = 3,50,000 cells per sample). As expected, these show that the estimate from individual devices is a good lower bound on the performance that can be attained from the device under conditions of noise, with the actual simulated performance being somewhere above that and below the minimal-noise performance.

## 3 Results

### 3.1 Implications for biological circuit engineering

Let us now consider how the engineering of biological circuits can be assisted by these models. We must, however, remember that having a strong predicted SNR for a circuit will not ensure that a biological circuit computes effectively, any more than using standard TTL components will ensure that an electronic circuit computes effectively: there are many other types of problems that also might interfere with the desired behavior. Importantly, though, having a strong SNR (both the overall circuit and the (SNR_dB_ at individual devices) does mean there is more margin for error in dealing with these other aspects of circuit engineering. Complementarily, an insufficient predicted SNR is a virtual guarantee that the circuit will not work. SNR analysis may thus be expected to be a useful tool for discriminating between possible circuit design alternatives.

As seen in the previous sections, in order to apply SNR analysis, it is necessary to have the following characterization data for each computational device:

- An input/output curve measured in compatible SI units, e.g., by means of the protocols in Beal et al. (2012, 2015), Davidsohn et al. (2015), and Kiani et al. (2014).^6^
- *σ_g_* for the device’s output in the (non-circuit) context in which the circuit is expected to operate.

To apply SNR analysis to a circuit requires the following additional information:

- The topology of the circuit, specifying the interconnections between device inputs and outputs.
- Input signal levels *σ_g_* and expression noise *σ_g_* (also implying input SNR).
- SNR requirements for the circuit output.

Given a library of characterized devices and a circuit specification, it is then possible to search for good candidate circuits. The best candidates should go beyond satisfying output SNR requirements and maximize output SNR, in order to have the most margin for dealing with other engineering difficulties. With a homogeneous library of devices with very similar behavior [e.g., as CRISPR-based repressors appear likely to provide (Kiani et al., 2014)], circuit viability can be determined by a straightforward application of the SNR analysis presented in the prior section and devices assigned arbitrarily. With a more heterogeneous library, [e.g., the TetR homolog library in Stanton et al. (2014)], different combinations of devices will have different properties, but the design problem should still be susceptible to efficient search with any number of well-established constrained-search methods (Russell and Norvig, 2003).

More important to the success of circuit engineering is the SNR characteristics of the devices in the library. The three circuit examples in the previous section are characteristic of three general qualitatively different “phases” of expected difficult in engineering biological circuits. These phases are predicted by considering the selection of devices from a library as a search process proposed by Beal (2014). The behavior of such a search process is critically affected by degree of coupling between design choices (i.e., the likelihood that two independent choices are incompatible), as has been well-established in complexity theory (Cheeseman et al., 1991; Hogg et al., 1996) and statistical physics (Krzakala and Kurchan, 2007; Dall’Asta et al., 2008; Zdeborová, 2008). In this case, the degree of coupling is determined by the likelihood of two devices having an output/input match with a high (SNR_dB_, leading to three qualitatively different expected engineering environments:

- *Difficult circuits*: when most biological devices in a library are either weak or poorly matched (e.g., having characteristics like Device B), it is difficult to discover a working combination of components even in the best circumstances.
  Engineering computational circuits using such devices is expected to be characterized by extensive and lengthy “tuning” and many failed attempts, since even small perturbations in device characteristics (e.g., from the biological operating context) can result in massive SNR losses.
- *Shallow circuits*: when many biological devices in a library have a large region of small negative SNR (e.g., having characteristics like Device C), it is easy to find acceptable matches, but there is still significant signal loss at every device.
  Engineering computational circuits using such devices is expected to be relatively simple for circuits up to a certain depth, because there is tolerance for small perturbations and many good candidates for working circuits. When the circuit requires more depth than can be readily attained while maintaining sufficient SNR, however, this strain raises the effective coupling and it will be extremely difficult to engineer an effective circuit of such depth, just as in the prior case.
- *Deep circuits*: when many biological devices in a library have a well-matched region with positive SNR (e.g., having characteristics like Device A), it is easy to find combinations of devices where signals do not degrade from layer to layer.
  Engineering computational circuits using such devices is expected to no longer be constrained by issues of computational efficacy: in principle, circuits of any depth and complexity can be readily engineered, and limits instead come from other aspects of the biological implementation, such as circuit delivery and demand on cellular resources.

Analysis of circuit and library SNR characteristics can determine which of these engineering environments we are operating in. Note, however, that there are no “hard” boundaries between phases: rather, as SNR characteristics improve, there is a gradual shift in the dominating engineering constraint from signal matching to signal degradation to non-signal constraints (with concomitant conclusions that can be drawn about the likely difficulty of circuit engineering). Unfortunately, knowing for certain if we are in trouble, while useful, does not actually make it any easier to engineer circuits. Quantification of SNR characteristics can, however, point to what target properties need to be achieved in order to move to a better engineering environment.

### 3.2 Prospects for deep circuit libraries

Given the widely observed difficulty of engineering biological systems [e.g., Kwok (2010)], it seems intuitive to guess that synthetic biology is currently operating in the “difficult circuits” regime. By applying SNR analysis to current high-efficacy device families, we can verify that this is actually the case. More importantly, however, we can also estimate approximately how far these device families are from the “shallow circuit” or “deep circuit” regimes, and what changes would be likely to allow them to attain those goals. When analyzing some properties of some device families, the relevant device characteristics are well enough known to allow rough quantification of requirements; in other cases, only qualitative conclusions can be drawn at present.

At present, there are several families of biological computational devices with the prospect of producing large numbers of universal logic devices with a high differential between output signal levels. The strongest current candidates are homolog mining, integrase logic, TALE and zinc finger repressors, and CRISPR-based repressors, each of which we discuss in detail. Other promising candidates include miRNA, aptamers, RNA-binding proteins, riboregulators, and protein/protein regulation, but all of these currently face various obstacles that mean they appear to be significantly farther away from providing large families of strong universal logic devices. As these technologies continue to mature, however, the same type of analysis presented in this section can be applied to them as well.

#### 3.2.1 Homolog Mining

**Figure 7:**
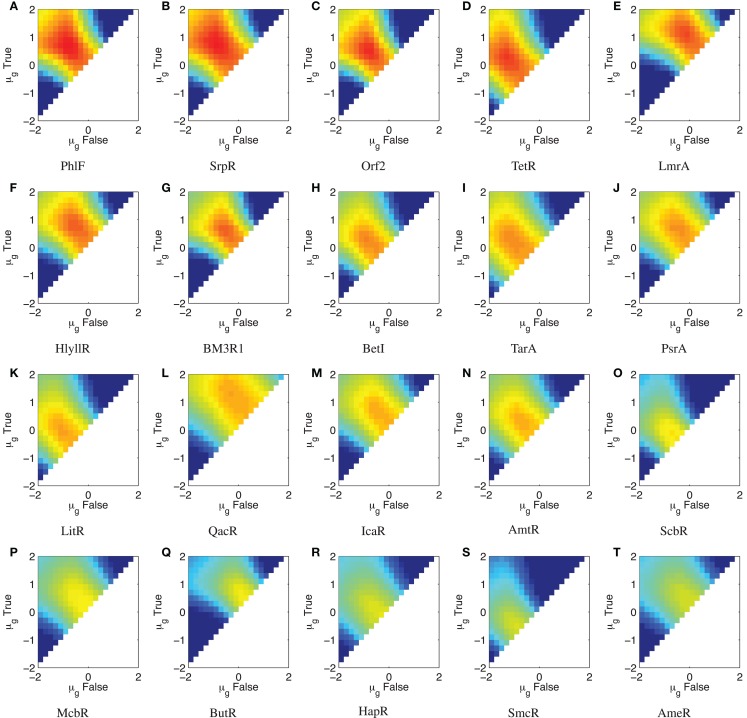
**Parameter scan of ΔSNR_dB_ for the TetR homolog library from Stanton et al. (2014) with σ_*g*_ = 2.0, sorted by maximum ΔSNR_dB_**. **(A–T)** show ΔSNR_dB_ for each device in the library, sorted in descending order of maximum ΔSNR_dB_. Colors use the same range as previously, from −20 to 5 dB. Note that the color scales are truncated at the lower end to provide better resolution in the upper range.

**Figure 8:**
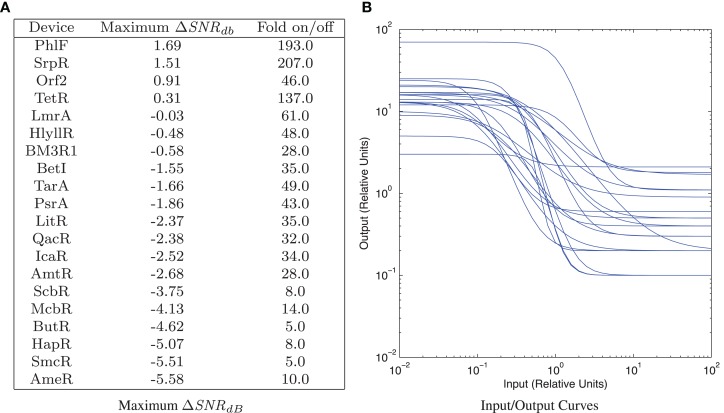
**TetR homolog library from Stanton et al. (2014): (A) maximum gate ΔSNR_dB_ with σ_*g*_ = 2.0, sorted by maximum ΔSNR_dB_**. **(B)** All input/output curves, computed from models, provided in Stanton et al. (2014).

The TetR repressor is a naturally occurring strong repressor that has been used successfully in many systems. Genomic mining for TetR homologs has produced a library of 20 orthogonal repressors, many of them with quite strong on/off ratios (Stanton et al., 2014). Each repressor has also been characterized with a high-resolution input/output curve (though only in relative units), and the models for these input/output curves are published in Stanton et al. (2014). Figure 7 shows parameter scans of (SNR_dB_ for a wide range of input level combinations for all 20 devices, using *σ_g_* = 2.0 as a conservatively low estimate of a typical value of bacterial expression noise, as estimated from the histograms reported in Stanton et al. (2014) and the noise values reported in Ozbudak et al. (2002). Parameters scans are performed as in Section 2.2 except shifted to the relative unit range of the devices (10^−2^–10^2^ relative units) and more coarsely, at five levels/decade. A summary of the results is given in Figure 8A, which lists the maximum (SNR_dB_ for each device, along with the on/off ratio reported in Stanton et al. (2014). Of the 20 reported gates, only 4 have the positive (SNR_dB_ needed that is a pre-requisite for deep circuits. Somewhere around another 5–10 are likely have sufficiently strong (SNR_dB_ for shallow circuits, given their relatively high amplification and moderate signal loss. Since the library is highly heterogeneous (Figure 8B), signal matching must be done on a circuit-by-circuit basis. One significant challenge that is clear from the input/output functions, however, is that few devices have an output *σ_g_*_,false_ low enough to achieve the optimal (SNR_dB_ input; the mismatches between devices can thus be expected to lower the effective (SNR_dB_ that can be achieved for any circuit.

Nevertheless, this library is the closest currently in existence to supporting deep circuits. Key targets for developing that capability are to further expand the library by additional mining, to calibrate the input/output curves to SI units, and to adjust the signal levels to better match, likely by decreasing output expression via 5′UTR modifications.

#### 3.2.2 Integrase Logic

Integrase logic gates, which operate by inverting segments of DNA, have been demonstrated to produce input/output curves with a very high amplification in their transition between high and low output (Bonnet et al., 2013). No model parameters were included in the publication, but the very steep slope of the curves makes it clear that these devices should have a high maximum (SNR_dB_. This is tempered, however, by a significant number of cells that do not change state, leading to a (SNR_dB_ that appears to be net negative rather than net positive.

At present, however, these integrase logic gates have quite poorly matched input and output signal levels. In addition, to date, very few have been demonstrated: it is reasonable to expect that many more might be discovered through homolog mining, though the availability of usable naturally occurring of orthogonal integrases is not yet clear. Key targets for expanding this technology into a library capable of deep computation are genomic mining to expand the number of devices, calibration of the input/output curves to SI units, and adjustment the signal levels to better match, likely by decreasing output expression via 5′UTR modifications.

#### 3.2.3 TALE and Zinc Finger Repressors

TALE proteins are a modular DNA-binding protein that can be engineered to bind to specific sequences with high specificity. Coupled with appropriately designed promoters, TALE proteins have been used to implement extensible libraries of strong promoters (Garg et al., 2012; Davidsohn et al., 2015; Li et al., 2015). TALE repressors can produce remarkably strong repression [measured at a maximum of nearly 5000-fold repression in Garg et al. (2012)]. Detailed input/output curves taken for TALE repressors in Davidsohn et al. (2015) and Li et al. (2015), however, have found a poor slope and uncertain match between input and output levels, implying a poor (SNR_dB_ for composed TALEs – consistent with the low input/output differential observed in the composite circuits investigated in that paper.

At present, TALEs are thus viable only for implementing very shallow circuits with low SNR. One likely path for increasing their potential depth is to increase repression strength by adjusting the synthetic promoter architectures used for TALE repressors. Given the level of deamplification observed in circuits in Davidsohn et al. (2015) and Li et al. (2015), an approximately 10-fold increase would likely be sufficient and may be attainable through this approach. Another possibility might be to heighten cooperativity (steepening the input/output curve) by changing the TALE to a fusion protein. Furthermore, the characterization in Davidsohn et al. (2015) was of transient rather than converged operation (i.e., fluorescence levels were still changing over time, rather than having reached a stable level of expression), and it is possible that TALE repressors may have a significantly steeper input/output curve when converged.

Zinc finger repressors are a very similar modular protein technology, which has also been demonstrated to produce strong orthogonal repressors [e.g., Khalil et al. (2012) and Lohmueller et al. (2012)]. No detailed input/output curves of these strong repressors have been produced to date, so obtaining input/output curves in SI units is the first key step to evaluating the viability of zinc finger repressors as a library. Given the similarity in promoter architectures used in the two technologies, however, it seems likely that they will face similar challenges to TALE repressors.

#### 3.2.4 CRISPR-Based Repressors

CRISPR-based repressors are a recent addition to the set of candidate libraries (Kiani et al., 2014), based on a protein that can be targeted with high specificity by a separately expressed sequence of guide RNA (gRNA). Like TALE and zinc finger repressors, they have showed very high repression strength, and may be significantly more homogeneous and easier to engineer with since the sequences are much shorter and do not involve any protein design. They have not yet had detailed input/output curves measured, however, and what characterization has been done to date has been of transient rather than converged behavior, as with TALE repressors.

For this family, the clear next step toward deep computation is to determine the SNR characteristics of the components, though this is complicated by their current use of a Pol III promoter to express gRNA, which is not compatible with the fluorescent proteins typically used for characterization. If the CRISPR-based repressors prove to have a steep slope in their converged behavior, then their SNR may already be sufficient for deep circuits; otherwise, they will likely require similar promoter engineering to TALE and zinc finger devices.

All told, we see that the current situation of synthetic biology is one of difficult circuit engineering. Even though some devices provide good SNR, there are not enough and there is not enough compatibility to reliably support engineering of either shallow or deep circuits. Other devices may also provide good SNR, but require characterization before this can be determined and, if true, effectively exploited. For all of these families of devices, however, the SNR approach identifies key targets for improvement that appear to be reasonable to aim for and that offer the prospect of enabling deep circuit engineering and the transformative capabilities that would imply.

## 4 Discussion of Contributions

This paper has developed methods for characterizing the efficacy of biological computing devices and circuits based on signal-to-noise ratio. This approach has the advantage of being firmly mathematically grounded in the fundamental definition of a signal, and can be applied using readily obtainable characterization data. This paper has also illustrated the use of SNR methods by applying them to analyze individual devices and predict the behavior of circuits in simulation, as well to develop a framework for SNR-based circuit engineering. Finally, a SNR-based analysis of current device libraries indicates that, while no library is yet sufficient to support deep biological circuits, several may be able to if particular targeted improvements can be realized.

One important direction for further development of this method is to extend it to a broader range of circuits and behaviors. Although this paper considered only static analysis of combinational Boolean logic circuits, there is no reason to think these cannot be extended to feedback circuits, analog circuits, and dynamic behavior of circuits. Another important direction is verification of the analysis and predictions made in this paper in the laboratory. This paper has also made specific predictions about particular targeted improvements to existing device libraries that should enable the engineering of deep biological circuits. In parallel with the progression of these other efforts, SNR methods are largely complementary to the methodologies considered by the many various prototype higher-level genetic circuit design tools [e.g., Myers et al. (2009), Beal et al. (2011), Bilitchenko et al. (2011), Marchisio and Stelling (2011), Yaman et al. (2012), and Huynh et al. (2013), to name a few], and have the potential to improve their operation by improving the metrics used by such tools for evaluating various design options. Investment to realize these improvements may thus have a revolutionary effect on the capabilities of synthetic biology, by enabling rapid engineering of complex computation and control circuits.

## Conflict of Interest Statement

The author declares that the research was conducted in the absence of any commercial or financial relationships that could be construed as a potential conflict of interest.

## Footnotes

^1^Note that the comparison of signal and noise distinguishes this discussion from prior investigations of gene expression noise in cells [e.g., Elowitz et al. (2002), Ozbudak et al. (2002), Rosenfeld et al. (2005), Bar-Even et al. (2006), and Friedman et al. (2006)]: these prior investigations characterize the characteristics of noise, but we cannot analyze the efficacy of computation without comparing such noise to an intended signal.

^2^MEFL units will be the unit of choice throughout this paper, as population histograms can be readily obtained experimentally in MEFL using protocols such as Beal et al. (2012), whereas other units such as concentration or number of molecules are much more difficult to obtain for large numbers of single cells at present. Using MEFL thus makes a simpler path for validation and application in the laboratory of the results presented.

^3^Such static analysis is expected to be a reasonable first approximation of cellular behavior with strong signals, given the apparent dominance of extrinsic vs. intrinsic noise under such conditions per Elowitz et al. (2002) and Rosenfeld et al. (2005).

^4^Hill equations simulate regulated production, not concentration, but in steady state (as we consider here) the two are linearly related by a constant.

^5^Correlations in expression noise can shift the results slightly, but the overall trends remain the same.

^6^Technically, relative units could be also used [e.g., RFUs, per Kelly et al. (2009)], but the lack of SI measurements means that it is much more difficult to debug any problems that arise, particularly with regards to differences between practitioners or laboratories.

## References

[B1] Adams D. S. Levin M. (2013). Endogenous voltage gradients as mediators of cell-cell communication: strategies for investigating bioelectrical signals during pattern formation. Cell Tissue Res. 352, 95–122. 10.1007/s00441-012-1329-4 22350846 PMC3869965

[B2] Bar-Even A. Paulsson J. Maheshri N. Carmi M. O’Shea E. Pilpel Y. (2006). Noise in protein expression scales with natural protein abundance. Nat. Genet. 38, 636–643. 10.1038/ng1807 16715097

[B3] Beal J. (2014). Bridging the gap: a roadmap to breaking the biological design barrier. Front. Bioeng. Biotechnol. 2:87. 10.3389/fbioe.2014.00087 25654077 PMC4299508

[B4] Beal J. Lu T. Weiss R. (2011). Automatic compilation from high-level biologically-oriented programming language to genetic regulatory networks. PLoS ONE 6:e22490. 10.1371/journal.pone.0022490 21850228 PMC3151252

[B5] Beal J. Wagner T. E. Kitada T. Azizgolshani O. Parker J. M. Densmore D. (2015). Model-driven engineering of gene expression from RNA replicons. ACS Synth. Biol. 4, 48–56. 10.1021/sb500173f 24877739

[B6] Beal J. Weiss R. Yaman F. Davidsohn N. Adler A. (2012). A Method for Fast, High-Precision Characterization of Synthetic Biology Devices. Technical Report MIT-CSAIL-TR-2012-008: MIT. Available at: http://hdl.handle.net/1721.1/69973.

[B7] Bilitchenko L. Liu A. Cheung S. Weeding E. Xia B. Leguia M. (2011). Eugene: a domain specific language for specifying and constraining synthetic biological parts, devices, and systems. PLoS ONE 6:e18882. 10.1371/journal.pone.0018882 21559524 PMC3084710

[B8] Bonnet J. Yin P. Ortiz M. E. Subsoontorn P. Endy D. (2013). Amplifying genetic logic gates. Science 340, 599–603. 10.1126/science.1232758 23539178

[B9] Canton B. Labno A. Endy D. (2008). Refinement and standardization of synthetic biological parts and devices. Nat. Biotechnol. 26, 787–793. 10.1038/nbt1413 18612302

[B10] Cheeseman P. Kanefsky B. Taylor W. M. (1991). “Where the really hard problems are,” in Proceedings of the 12th International Joint Conference on Artificial Intelligence, Vol. 1 (San Francisco, CA: Morgan Kaufmann Publishers Inc.), 131–337.

[B11] Dall’Asta L. Ramezanpour A. Zecchina R. (2008). Entropy landscape and non-gibbs solutions in constraint satisfaction problems. Phys. Rev. E Stat. Nonlin. Soft Matter Phys. 77, 031118. 10.1103/PhysRevE.77.031118 18517340

[B12] Danino T. Mondragon-Palomino O. Tsimring L. Hasty J. (2009). A synchronized quorum of genetic clocks. Nature 463, 326–330. 10.1038/nature08753 20090747 PMC2838179

[B13] Davidsohn N. Beal J. Kiani S. Adler A. Yaman F. Li Y. (2015). Accurate predictions of genetic circuit behavior from part characterization and modular composition. ACS Synth. Biol. 4, 673–681. 10.1021/sb500263b 25369267

[B14] Ellis T. Wang X. Collins J. (2009). Diversity-based, model-guided construction of synthetic gene networks with predicted functions. Nat. Biotechnol. 27, 465–471. 10.1038/nbt.1536 19377462 PMC2680460

[B15] Elowitz M. Leibler S. (2000). A synthetic oscillatory network of transcriptional regulators. Nature 403, 335–338. 10.1038/35002125 10659856

[B16] Elowitz M. B. Levine A. J. Siggia E. D. Swain P. S. (2002). Stochastic gene expression in a single cell. Science 297, 1183–1186. 10.1126/science.1070919 12183631

[B17] Friedman N. Cai L. Xie X. S. (2006). Linking stochastic dynamics to population distribution: an analytical framework of gene expression. Phys. Rev. Lett. 97, 168302. 10.1103/PhysRevLett.97.168302 17155441

[B18] Gardner T. S. Cantor C. R. Collins J. J. (2000). Construction of a genetic toggle switch in escherichia coli. Nature 403, 339–342. 10.1038/35002131 10659857

[B19] Garg A. Lohmueller J. J. Silver P. A. Armel T. Z. (2012). Engineering synthetic tal effectors with orthogonal target sites. Nucleic Acids Res. 40, 7584–7595. 10.1093/nar/gks404 22581776 PMC3424557

[B20] Hill A. V. (1910). The possible effects of the aggregation of the molecules of haemoglobin on its dissociation curves. J. Physiol. 40, 4–7. 10.1371/journal.pone.0041098 22844429 PMC3402542

[B21] Hogg T. Huberman B. A. Williams C. P. (1996). Phase transitions and the search problem. Artif. Intell. 81, 1–15. 10.1016/0004-3702(95)00050-X

[B22] Huynh L. Tsoukalas A. Köppe M. Tagkopoulos I. (2013). Sbrome: a scalable optimization and module matching framework for automated biosystems design. ACS Synth. Biol. 2, 263–273. 10.1021/sb300095m 23654271

[B23] Kelly J. R. Rubin A. J. Davis J. H. Ajo-Franklin C. M. Cumbers J. Czar M. J. (2009). Measuring the activity of biobrick promoters using an in vivo reference standard. J. Biol. Eng. 3, 4. 10.1186/1754-1611-3-4 19298678 PMC2683166

[B24] Keung A. J. Bashor C. J. Kiriakov S. Collins J. J. Khalil A. S. (2014). Using targeted chromatin regulators to engineer combinatorial and spatial transcriptional regulation. Cell 158, 110–120. 10.1016/j.cell.2014.04.047 24995982 PMC4110908

[B25] Khalil A. S. Lu T. K. Bashor C. J. Ramirez C. L. Pyenson N. C. Joung J. K. (2012). A synthetic biology framework for programming eukaryotic transcription functions. Cell 150, 647–658. 10.1016/j.cell.2012.05.045 22863014 PMC3653585

[B26] Kiani S. Beal J. Ebrahimkhani M. R. Huh J. Hall R. N. Xie Z. (2014). Crispr transcriptional repression devices and layered circuits in mammalian cells. Nat. Methods 11, 723–726. 10.1038/nmeth.2969 24797424 PMC4228775

[B27] Kim B. Lin M. Z. (2013). Optobiology: optical control of biological processes via protein engineering. Biochem. Soc. Trans. 41, 1183–1188. 10.1042/BST20130150 24059506 PMC4076147

[B28] Knight T. F. Jr. Sussman G. J. (1998). “Cellular gate technology,” in Unconventional Models of Computation (Berlin: Springer-Verlag), 257–272.

[B29] Krzakala F. Kurchan J. (2007). Landscape analysis of constraint satisfaction problems. Phys. Rev. E Stat. Nonlin. Soft Matter Phys. 76, 021122. 10.1103/PhysRevE.76.021122 17930021

[B30] Kwok R. (2010). Five hard truths for synthetic biology. Nature 463, 288–290. 10.1038/463288a 20090726

[B31] Li Y. Jiang Y. Chen H. Liao W. Li Z. Weiss R. (2015). Modular construction of mammalian gene circuits using tale transcriptional repressors. Nat. Chem. Biol. 11, 207–213. 10.1038/nchembio.1736 25643171 PMC4333066

[B32] Linshiz G. Stawski N. Poust S. Bi C. Keasling J. D. Hillson N. J. (2012). Par-par laboratory automation platform. ACS Synth. Biol. 2, 216–222. 10.1021/sb300075t 23654257

[B33] Lohmueller J. J. Armel T. Z. Silver P. A. (2012). A tunable zinc finger-based framework for boolean logic computation in mammalian cells. Nucleic Acids Res. 40, 5180–5187. 10.1093/nar/gks142 22323524 PMC3367183

[B34] Lou C. Stanton B. Chen Y.-J. Munsky B. Voigt C. A. (2012). Ribozyme-based insulator parts buffer synthetic circuits from genetic context. Nat. Biotechnol. 30, 1137–1142. 10.1038/nbt.2401 23034349 PMC3914141

[B35] Marchisio M. A. Stelling J. (2011). Automatic design of digital synthetic gene circuits. PLoS Comput. Biol. 7:e1001083. 10.1371/journal.pcbi.1001083 21399700 PMC3048778

[B36] Moon T. Lou C. Tamsir A. Stanton B. Voigt C. (2012). Genetic programs constructed from layered logic gates in single cells. Nature 491, 249–253. 10.1038/nature11516 23041931 PMC3904217

[B37] Mutalik V. K. Guimaraes J. C. Cambray G. Lam C. Christoffersen M. J. Mai Q.-A. (2013). Precise and reliable gene expression via standard transcription and translation initiation elements. Nat. Methods 10, 354–360. 10.1038/nmeth.2404 23474465

[B38] Myers C. J. Barker N. Jones K. Kuwahara H. Madsen C. Nguyen N.-P. D. (2009). ibiosim: a tool for the analysis and design of genetic circuits. Bioinformatics 25, 2848–2849. 10.1093/bioinformatics/btp457 19628507

[B39] Oppenheim A. V. Willsky A. S. (1997). Signals and Systems. Upper Saddle River, NJ: Prentice-Hall.

[B40] Ozbudak E. M. Thattai M. Kurtser I. Grossman A. D. van Oudenaarden A. (2002). Regulation of noise in the expression of a single gene. Nat. Genet. 31, 69–73. 10.1038/ng869 11967532

[B41] Purnick P. E. M. Weiss R. (2009). The second wave of synthetic biology: from modules to systems. Nat. Rev. Mol. Cell Biol. 10, 410–422. 10.1038/nrm2698 19461664

[B42] Rosenfeld N. Young J. W. Alon U. Swain P. S. Elowitz M. B. (2005). Gene regulation at the single-cell level. Science 307, 1962–1965. 10.1126/science.1106914 15790856

[B43] Russell S. J. Norvig P. (2003). Artificial Intelligence: A Modern Approach, 2 Edn New York, NY: Pearson Education.

[B44] Stanton B. Nielsen A. Tamsir A. Clancy K. Peterson T. Voigt C. (2014). Genomic mining of prokaryotic repressors for orthogonal logic gates. Nat. Chem. Biol. 10, 99–105. 10.1038/nchembio.1411 24316737 PMC4165527

[B45] Weber E. Engler C. Gruetzner R. Werner S. Marillonnet S. (2011). A modular cloning system for standardized assembly of multigene constructs. PLoS ONE 6:e16765. 10.1371/journal.pone.0016765 21364738 PMC3041749

[B46] Weiss R. (2001). Cellular Computation and Communications using Engineered Genetic Regulatory Networks. Ph.D. thesis, MIT, Cambridge, MA.

[B47] Yaman F. Bhatia S. Adler A. Densmore D. Beal J. (2012). Automated selection of synthetic biology parts for genetic regulatory networks. ACS Synth. Biol. 1, 332–344. 10.1021/sb300032y 23651287

[B48] Zdeborová L. (2008). Statistical Physics of Hard Optimization Problems. *arXiv* arXiv:0806.4112. Available at: http://arxiv.org/abs/0806.4112

